# Comparative analyses of complete chloroplast genomes reveal interspecific difference and intraspecific variation of *Tripterygium* genus

**DOI:** 10.3389/fpls.2023.1288943

**Published:** 2024-01-09

**Authors:** Kai-Ling Xu, Zhong-Mou Zhang, Wen-Liang Fang, Ya-Dan Wang, Hong-Yu Jin, Feng Wei, Shuang-Cheng Ma

**Affiliations:** ^1^ Institute for Control of Chinese Traditional Medicine and Ethnic Medicine, National Institutes for Food and Drug Control, Beijing, China; ^2^ School of Traditional Chinese Pharmacy, China Pharmaceutical University, Nanjing, China

**Keywords:** *Tripterygium wilfordii*, *Tripterygium hypoglaucum*, chloroplast genome, genome structure, phylogenetic analysis, hypervariable regions

## Abstract

The genus *Tripterygium* was of great medicinal value and attracted much attention on the taxonomic study using morphological and molecular methods. In this study, we assembled 12 chloroplast genomes of *Tripterygium* to reveal interspecific difference and intraspecific variation. The sequence length (156,692–157,061 bp) and structure of *Tripterygium* were conserved. Comparative analyses presented abundant variable regions for further study. Meanwhile, we determined the *ndhB* gene under positive selection through adaptive evolution analysis. And the phylogenetic analyses based on 15 chloroplast genomes supported the monophyly of *Tripterygium hypoglaucum* and the potential sister relationship between *Tripterygium wilfordii* and *Tripterygium regelii*. Molecular dating analysis indicated that the divergence time within *Tripterygium* was approximately 5.99 Ma (95% HPD = 3.11–8.68 Ma). The results in our study provided new insights into the taxonomy, evolution process, and phylogenetic construction of *Tripterygium* using complete plastid genomes.

## 1 Introduction

The genus *Tripterygium* (Celastraceae) comprises three species, *Tripterygium wilfordii*, *Tripterygium hypoglaucum*, and *Tripterygium regelii*, which were widely distributed in central and eastern Asia. As a renowned traditional medicine for treating arthritis, swelling, autoimmune disease, and diabetic nephropathy (Tao et al., 1991; Ge et al., 2013; Wang et al., 2016), it has attracted extensive attention from scholars, especially phytochemists. More than 500 compounds have been isolated and identified, including triptolide, a bioactive diterpene triepoxide that has been studied for decades (Lv et al., 2019; Tong et al., 2021). However, the boundaries of interspecific plant morphology, such as leaf, flower, and samara characteristics, are blurred (Brinker et al., 2007). The root of *T. wilfordii* and *T. hypoglaucum* was used as a medicinal part and widely applied in various commercial pharmaceutical preparations; their microscopic and morphological characteristics were difficult to distinguish, leading to potential issues in drug quality control. Differences in chemical composition of these three species have been confirmed by HPLC, RRLC-ESI-MS^n^, and PCA analyses (Guo et al., 2014; Chen et al., 2017). However, molecular phylogenetic studies using RAPD, 5S rDNA, ITS, or the combination of these DNA regions, presented different perspectives. Some authors considered that *T*. *wilfordii* and *T. hypoglaucum* were potentially conspecific, while *T*. *regelii* was recognized as a separate species (Liu et al., 2007; Law et al., 2011; Zhang et al., 2016; Ma et al., 2017). Therefore, it is urgently needed to develop effective methods to study phylogenetic relationships and provide taxonomic clarity of this genus.

With the popularization and cost reduction of next-generation sequencing technology, comparative analyses of complete plastid genomes were increasingly being applied for phylogenetic studies. Chloroplasts, which are mainly found in plant cells, are essential organelles that conduct photosynthesis, carbon fixation, and other fundamental intermediary metabolic reactions (Xiong et al., 2009). The structure of chloroplast genomes was divided into four parts, two copies of inverted repeat (IR) regions, a large single-copy (LSC) region, and a small single-copy (SSC) region (Palmer, 1985; Xu et al., 2023). The chloroplast genome was highly conserved (typically between 120 and 220 kb) and had relatively moderate evolutionary rate, which could provide vital information for the classification and phylogenetic relationship construction among species (Yan et al., 2022).

Currently, the classification and phylogeny studies of *Tripterygium* are only based on short DNA regions, lacking research based on complete chloroplast genomes, making it difficult to fully resolve the taxonomical controversies of this genus. Therefore, in this study, 12 samples of genus *Tripterygium* distributed in the main producing areas of China were collected. Comparative analyses using complete chloroplast genomes obtained and annotated in our study were conducted to reveal the interspecific difference and intraspecific variation of *Tripterygium* genus. And analyses of adaptive evolution, molecular divergence time, and phylogeny were used to study the evolution process and phylogenetic relationships within *Tripterygium*.

## 2 Materials and methods

### 2.1 Plant materials, DNA extraction and sequencing

As *T*. *wilfordii* and *T. hypoglaucum* were usually for clinical use and the classification between them was controversial, fresh leaves of four *T*. *wilfordii* and eight *T. hypoglaucum* were collected, and the detailed location information was shown in **Supplementary Table S1**. Moreover, a sequence of *T*. *regelii* was mined from GenBank for comparative analyses. Total genomic DNA was extracted using TianGen DP 305 Plant Genomic DNA Kit in accordance with the instruction. DNA quality was assessed using a NanoDrop spectrophotometer (Thermo Scientific, Carlsbad, CA, USA) and Agilent 5400 Fragment Analyzer. The DNA samples were then sent to Novogene (Novogene Bioinformatics Technology Co., Ltd., Beijing, China) for library construction. The qualified libraries were pooled and sequenced on Illumina platforms with PE150 strategy. Fastp v.0.23.1 (Chen et al., 2018) was used to control quality and remove sequencing adaptors and low-quality bases. Finally, over 2 G clean data of all samples were prepared for further study.

### 2.2 Chloroplast genome assembly and annotation

Filtered reads were assembled by SPAdes v3.13.1 genome assembler (Bankevich et al., 2012) with kmer sizes of 35, 55, 77, and 127. The MUMmer program (Kurtz et al., 2004) was used to compare the assembled contigs with the reference genome (MN624264). All chloroplast genomes were reconstructed by reference-based assembly. Annotation was conducted by CPGAVAS2 (Shi et al., 2019) and checked manually. Finally, the chloroplast genomes were plotted by Chloroplot (Zheng et al., 2020). All of the chloroplast genomes and annotations were uploaded to GenBank and assigned the accession numbers OR426549-52 and OR426554-60 (**Table 1**).

**Table 1:** Characteristics of complete chloroplast genomes.

Species	Sample ID	Accession numbers	Length (bp)	GC content (%)	LSC (bp)	IR (bp)	SSC (bp)
*T*. *wilfordii*	ZJ	OR426549	156,692	37.47	85,402	26,461	18,368
	FJ1	OR426550	156,692	37.47	85,402	26,461	18,368
	FJ2	OR426551	156,699	37.47	85,409	26,461	18,368
	HB2	OR426552	156,692	37.47	85,402	26,461	18,368
*T. hypoglaucum*	GX1	OR426554	156,998	37.44	85,362	26,594	18,448
	GX2	OR426555	157,061	37.45	85,424	26,602	18,433
	YN1	OR426556	156,936	37.48	85,324	26,589	18,434
	YN2	OR426556	156,936	37.48	85,324	26,589	18,434
	SC1	OR426557	157,055	37.46	85,412	26,592	18,459
	SC2	OR426558	157,022	37.46	85,404	26,592	18,434
	SC3	OR426559	157,051	37.47	85,409	26,592	18,458
	HB1	OR426560	157,054	37.44	85,407	26,592	18,463
*T*. *regelii*	DB	MN624266	159,144	37.56	87,556	26,592	18,404

### 2.3 Comparative analyses of genome structure

The boundaries of LSC, SSC, and IR regions in all of the *Tripterygium* chloroplast genomes were compared by CPJSdraw (Li et al., 2023). The mVISTA (Frazer et al., 2001) was applied to visualize the variations among these genomes in Shuffle-LAGAN mode. The dispersed and palindromic repeats were determined by REPuter (Kurtz et al., 2001) with Hamming distance set to 1 and the minimum repeat size set to 30 bp. Based on the default parameters, the online Tandem Repeats Finder (Benson, 1999) program was applied for tandem repeat calculation, but the minimum alignment score was set to 80. Simple sequence repeats (SSRs) were detected using MISA Perl script (Thiel et al., 2003), the parameter of which was set as thresholds of 10 repeat units for mono-, six repeat units for di-, four repeat units for tri- and tetra-, and three repeat units for penta- and hexanucleotide SSRs.

The evolution rate was analyzed according to the non-synonymous (dN), synonymous (dS), and their ratio (*ω* = dN/dS) after extracting the common functional protein-coding sequences in *Tripterygium* (sequences represented by FJ1, YN2, and DB). The positive selection sites were determined by program PAML4.9 (Yang, 2007) with site-specific model implemented in the codeml package and Bayes Empirical Bayes (BEB) analysis. And the parameters were set as previously reported (Fan et al., 2018). Candidate sites for positive selection [*p*_(_ *_ω_*_> 1)_ > 0.95] were selected for two Likelihood ratio tests (M1 vs. M2 and M7 vs. M8).

The nucleotide diversity (Pi) was calculated *via* the DnaSP (Rozas et al.) program after the clusters of coding and noncoding regions were extracted. And the possible DNA rearrangement among chloroplast genomes was detected by Mauve plugin in GENEIOUS (Darling et al., 2010).

### 2.4 Phylogenetic analyses

Phylogenetic topology of *Tripterygium* was reconstructed using Maximum likelihood (ML) and Bayesian inference (BI) methods. Fifteen complete chloroplast genomes were used for the study of phylogenetic relationships, including 13 sequences of genus *Tripterygium*. Sequences of *Celastrus orbiculatus* (MW316708) and *Celastrus stylosus* (MZ508373) were chosen as outgroups to root the tree. All of the chloroplast genomes were aligned by MAFFT v.7.520 (Katoh and Standley, 2013) and trimmed by trimAI v.1.2 (Capella-Gutiérrez et al., 2009) with the option of automated1. ML analyses were performed using IQ-TREE v.1.6.12 (Nguyen et al., 2015) with 10,000 bootstrap replications, in which the best fitting model was selected (Kalyaanamoorthy et al., 2017). BI analyses were conducted by MrBayes 3.2.7 (Ronquist et al., 2012). The best-fit model (GTR+I+G) was selected by Akaike information criterion (AIC) in MrModeltest 2.4 (Guo et al., 2022). And the BI analyses were run for 2,000,000 generations and sampled every 100 generation. The first 25% of trees were discarded as burn-ins. And the program Tracer 1.7.2 (Rambaut et al., 2018) was used to determine the effective sample size (>200). Finally, the obtained trees were visualized *via* iTOL (Letunic and Bork, 2021).

### 2.5 Divergence time estimation

BEAST v.1.10.4 (Drummond et al., 2012) was applied to estimate the divergence times with Yule process speciation prior and uncorrelated relaxed clock. The split between *Tripterygium* and the outgroup *C. orbiculatus* was constrained to be 27 Ma, and 5.46–11.6 Ma was set for the split within *Tripterygium* (Ma et al., 2017). MCMC chains were run for 1,000,000,000 generations and sampled every 1,000 generations. Tracer 1.7.2 (Rambaut et al., 2018) was used to evaluate the convergence according to the effective sample size (ESS > 200) with the first 10% discarded as burn-ins. TreeAnnotator v.1.10.1 (Drummond et al., 2012) was used to summarize and annotate the tree with a burn-in of 25%. Finally, the tree was visualized in the program Figtree v.1.4.4 (http://tree.bio.ed.ac.uk/) with 95% highest posterior density (95% HPD).

## 3 Results

### 3.1 Structure features of chloroplast genomes in the genus *Tripterygium*

**Figure 1:**
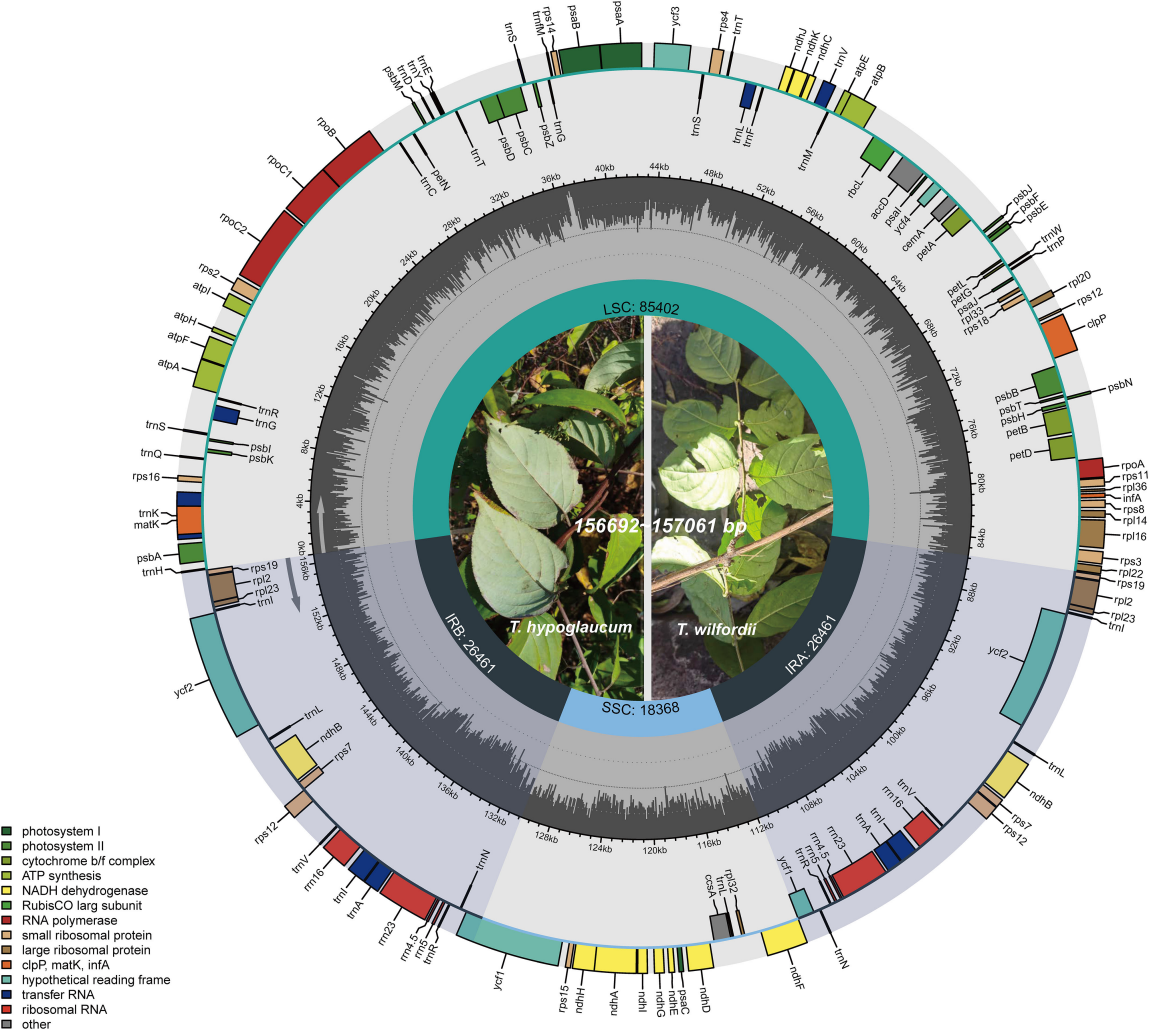
Chloroplast genome gene map of *Tripterygium* (represented by ZJ).

The length of 12 assembled chloroplast genomes (**Figure 1**; **Table 1**) ranged from 156,692 bp (*T*. *wilfordii*, ZJ, FJ1, and HB2) to 157,061 bp (*T. hypoglaucum*, GX2), while the length of *T*. *regelii* was 159,144 bp (MN624266). As the plastid genome of *T. hypoglaucum* collected from Ziwu Town, Yunnan Province, was consistent with the plastid genome collected from Donghua Town, they are collectively named YN. Moreover, the sequence of *T. wilfordii* sample FJ1 was identical to HB2. All of the assembled chloroplast genomes have a typical quadripartite structure with an LSC region (85,324–87.556 bp), an SSC region (18,368–18,463 bp), and a pair of IR regions (26,461–26,602 bp). In addition, the whole guanine-cytosine (GC) contents ranged from 37.44% to 37.56%. All of the complete *Tripterygium* genomes contained 112 unique annotated genes, including 78 protein-coding genes, 4 rRNA genes, and 30 tRNA genes (**Supplementary Table S2**).

### 3.2 Comparative chloroplast genomic analyses of the genus *Tripterygium*

**Figure 2:**
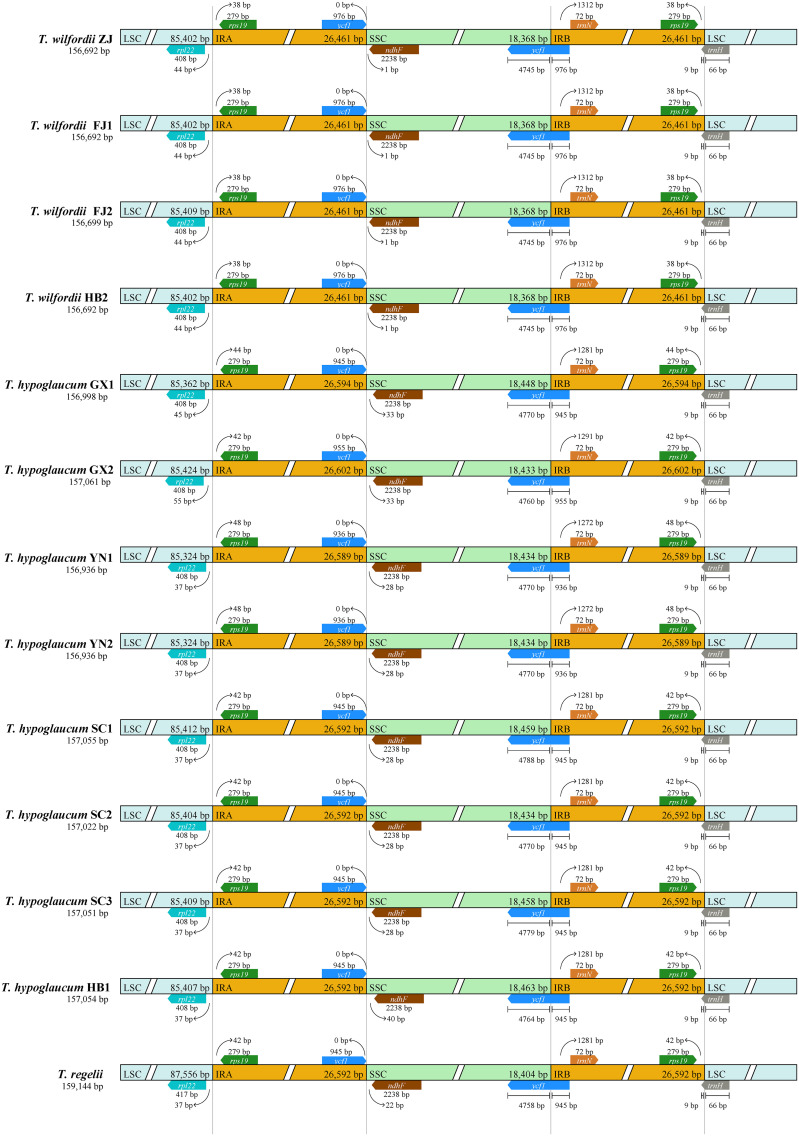
Boundary comparison of LSC, SSC, and IR regions among the *Tripterygium* chloroplast genomes.

The IR/SC boundaries of *Tripterygium* were illustrated and compared to address IR expansion and contraction (**Figure 2**). As the result showed, the *Tripterygium* chloroplast genomes were conserved. The boundary of SSC/IRB fell within the *ycf1* gene. And the SSC/IRA junctions were located between the pseudogene *ycf1* and gene *ndhF*, while the LSC/IRA junctions were located between the gene *rpl22* and *rps19*. In particular, all of the gene *trnH* of *Tripterygium* crossed the LSC and IRB boundary with 66 bp in LSC. It seemed that the variation of SSC/IRA boundary could be categorizable into three classes like the species within this genus. The interval between *ndhF* and the SSC/IRA boundary was 1 bp in *T. wilfordii*. However, a 28–40-bp interval in SSC was exhibited in *T. hypoglaucum*, with the neighboring distributions having the same intervals, such as the samples collected from Yunan (YN) and Sichuan province (SC1–3).

**Figure 3:**
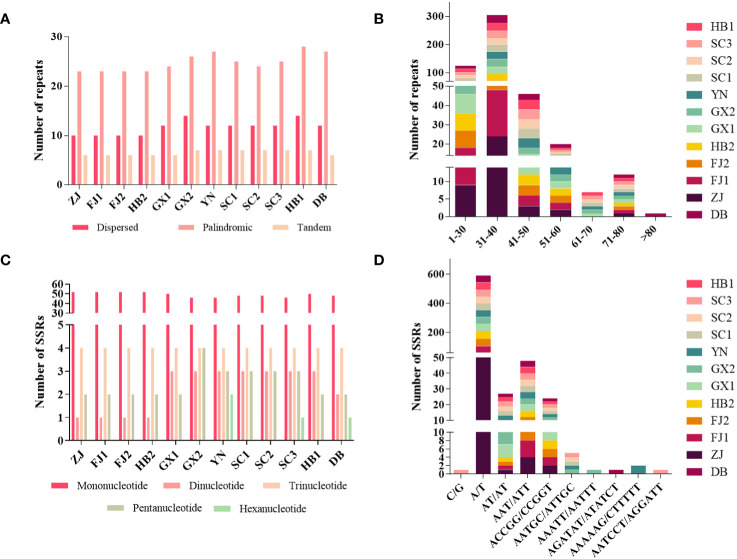
Analyses of repeat elements in *Tripterygium*: **(A)** numbers of dispersed, palindromic and tandem repeats; **(B)** distribution of repeat motifs size; **(C)** numbers of different repeat SSRs types; **(D)** numbers of different SSR class types.

**Figure 4:**
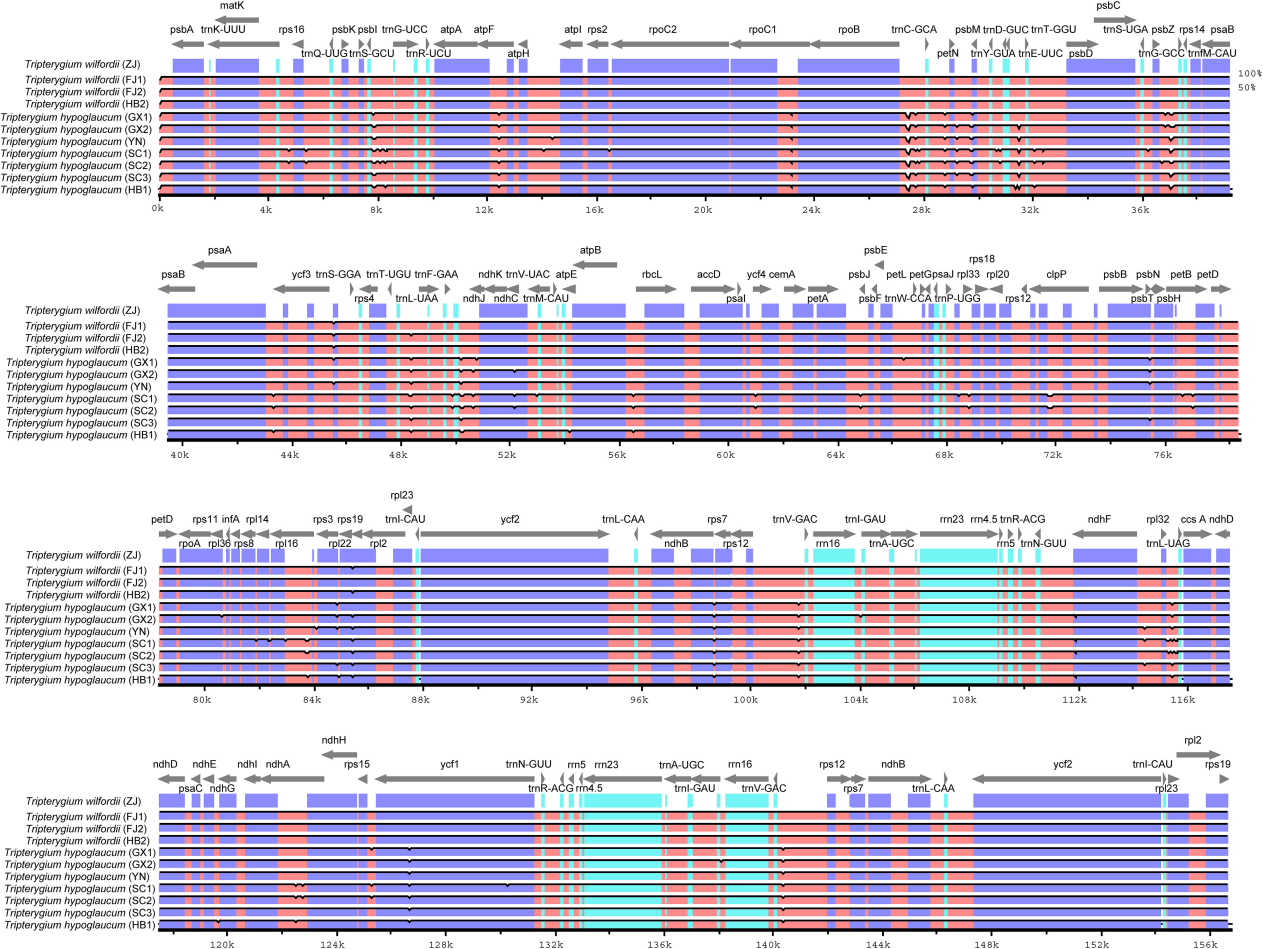
Alignment of the complete chloroplast genomes obtained. Gray arrows above the alignment indicate gene orientation. The right Y-axis indicated similarity in the 50%–100% range. Purple bars represent exons, blue bars represent UTRs, and pink bars represent CNS.

**Figure 5:**
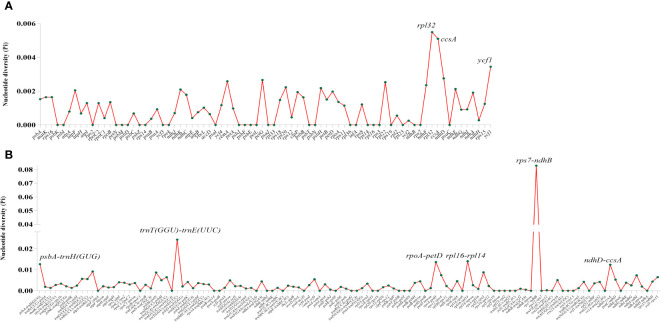
Nucleotide diversity of coding regions **(A)** and noncoding regions **(B)**.

Three kinds of repeat elements, dispersed, palindromic, and tandem repeats, were calculated in this work. Among these types of *Tripterygium*, the palindromic repeats accounted for the largest proportion, followed by the dispersed repeats, and the tandem repeats accounted for the least (**Figure 3A**). Meanwhile, the number and distribution of all repeat types in *T. wilfordii* were the same, with 23 palindromic repeats, 10 dispersed repeats, and 6 tandem repeats, and could be distinguished from *T. hypoglaucum* and *T*. *regelii*. And the repeat motif size of *Tripterygium* was mainly concentrated in the range of 31–40 bp, followed by 1–30 bp (**Figure 3B**). In particular, a unique 63-bp repeat unit was present in *T. hypoglaucum* collected from different regions. Given the high polymorphism of SSRs in the species, they have been widely used for the evolutionary and ecological research and considered as important molecular markers to study intraspecific variation. The total number of SSRs in *Tripterygium* ranged from 57 to 59. Among these SSRs, the mononucleotide repeats were the most abundant (ranged from 46 to 52), followed by the trinucleotide repeats, the whole of which were 4 in *Tripterygium* (**Figure 3C**). Hexanucleotide only occurred in samples YN, SC3, and DB, while dinucleotide occurred in all samples and could be divided into three classes. The number of dinucleotide repeats was 1 in *T. wilfordii*, 3 in *T. hypoglaucum*, and 2 in *T*. *regelii*, respectively. As **Figure 3D** showed, A/T repeats were abundant, while G/C repeats only existed in sample SC3. All of the samples had the A/T, AT/AT, AAT/ATT, and ACCGG/CCGGT repeats. In addition, the numbers of AAT/ATT and ACCGG/CCGGT repeats were identical in all chloroplast genomes of *Tripterygium*, which were 4 and 2, respectively. The divergence of assembled and annotated sequences was compared using the sample ZJ as a reference (**Figure 4**). It was exhibited that all of the plastid genomes were relatively conserved. Overall, the noncoding regions were more divergent than coding regions. Intergenic spacer regions, such as *trnG(UCC)-trnS(GCU)*, *trnC(GCA)-rpoB*, *trnG(GCC)-psbZ*, *ndhJ-trnF(GAA)*, and *trnL(UAG)-rpl32*, showed relatively high variability according to the results of multiple alignment, which could be studied as a potential molecular marker to distinguish *T. wilfordii* and *T. hypoglaucum* because of the significant difference in sequence length. In addition, the nucleotide diversity (Pi) of coding regions and non-coding regions was calculated (**Figures 5A, B**). In the coding regions, the genes *rpl32*, *ccsA* and *ycf1* showed the most abundant variation (Pi > 0.003), while in the intergenic spacer regions, *psbA-trnH(GUG)*, *trnT(GGU)-trnE(UUC)*, *rpoA-petD*, *rpl16-rpl14*, *rps7-ndhB* and *ndhD-ccsA* had higher Pi values (Pi > 0.01). These variable regions mentioned above could be useful to find potential DNA barcodes for *Tripterygium* species authentication. In addition, no genomic rearrangements have been detected in chloroplast genomes (**Supplementary Figure 1**).

In this study, 75 protein-coding genes extracted from *T. wilfordii* (sample FJ1), *T. hypoglaucum* (sample YN2), and *T*. *regelii* (sample DB) were used for the adaptive evolution analysis. Positively selected sites were found in *atpA*, *rpl20*, *rpoA*, *ccsA*, *matK*, and *ndhB* genes (**Supplementary Table S3**). Meanwhile, there are eight positively selected sites in the *ccsA* gene under M2 and M8 models, two in the *atpA* gene, three in *matK* gene, and one in others. However, two LRTs (M1 vs. M2 and M7 vs. M8) only supported that the *ndhB* gene (**Supplementary Table S4**) had the positively selected codon sites (*p* < 0.05).

### 3.3 Phylogenetic analyses

**Figure 6:**
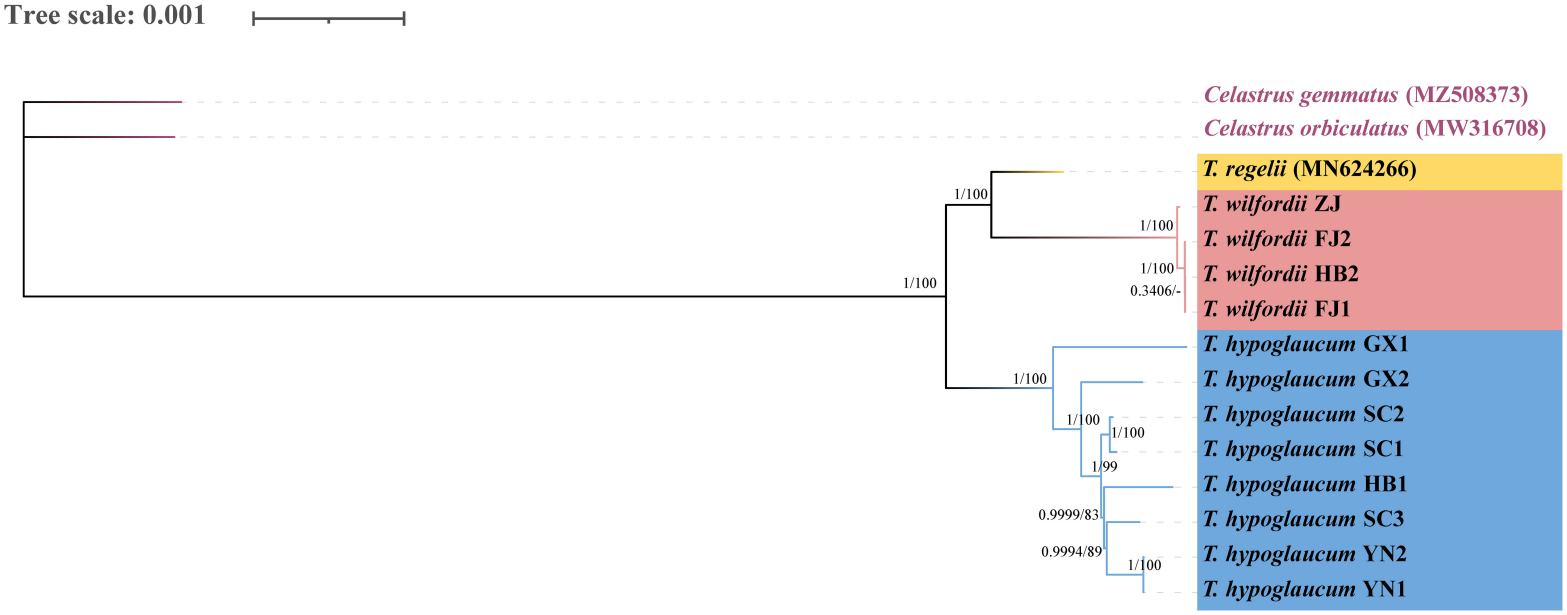
Phylogenetic tree reconstructed based on the complete chloroplast genomes by Bayesian interference (BI) and maximum likelihood (ML). Numbers represent BI posterior probabilities (PP) and ML bootstrap values (BS), respectively.

The phylogenetic relationship was reconstructed based on 15 plastid genomes, including adjacent genera *C. orbiculatus* (MW316708) and *C. stylosus* (MZ508373), which were set as outgroups to root the tree. Among the whole sequences, 154,954 constant sites, with 1,278 parsimony-informative characters were used for alignment. The topologies of the ML and BI trees based on the chloroplast genomes were consistent (**Figure 6**). Two strongly supported clades (PP/BS = 1/100) were observed, which showed that *T. hypoglaucum* was monophyletic, whereas *T. wilfordii* was nested with *T. regelii*. The subclades of *T. wilfordii* and *T. regelii* could be further distinguished because of the strong support (PP/BS = 1/100). This tree confirmed the classification of *Tripterygium* and indicated that *T. wilfordii* could be distinguished from *T. hypoglaucum*.

### 3.4 Molecular dating

**Figure 7:**
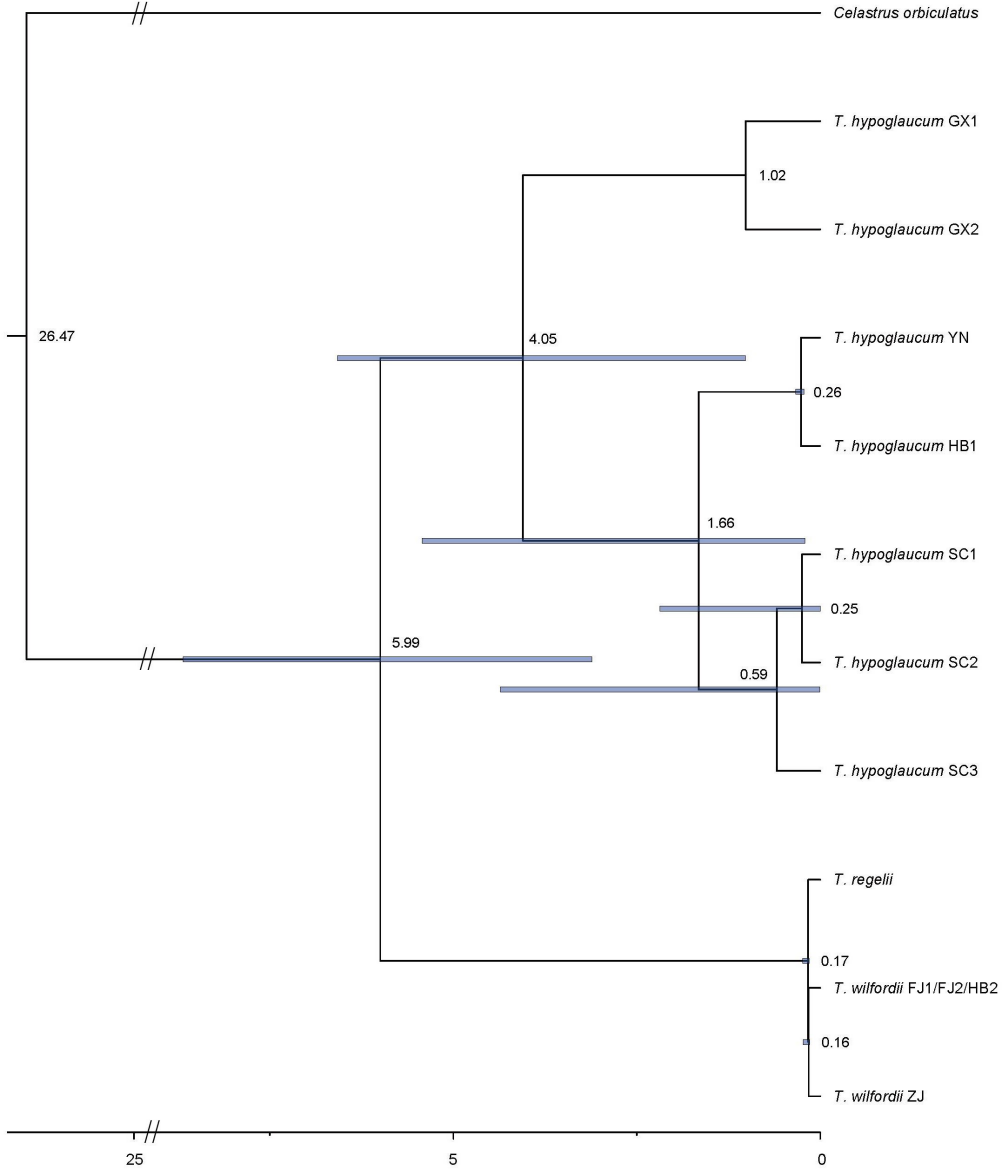
Molecular dating of the genus *Tripterygium* based on the CDS region in chloroplast genomes.

We estimated the divergence time of the genus *Tripterygium* based on the CDS of protein-coding genes in the complete chloroplast genomes. As the coding sequences (CDSs) extracted from samples YN1 and YN2, as well as FJ1, FJ2, and HB2, were the same, divergence time estimation has not been conducted separately. Finally, the result (**Figure 7**) showed that the divergence between *T. hypoglaucum* and the cluster of *T. wilfordii* and *T. regelii* was estimated to occur in Miocene (5.99 Ma, 95% HPD = 3.11–8.68 Ma). In addition, the divergence time of *T. wilfordii* and *T. regelii* probably originated at 0.17 Ma (in the late Pleistocene, 95% HPD = 0.15–0.24 Ma), later than that of *T. hypoglaucum* (4.05 Ma, in the Plicocene, 95% HPD = 1.02–6.58 Ma). Interestingly, within *T. hypoglaucum*, except for the two samples collected from Guangxi province, all others arose at 1.66 Ma (95% HPD = 0.21–5.42 Ma).

## 4 Discussion

The taxonomic studies of the genus *Tripterygium* have been controversial in recent decades, especially in the distinction between *T. wilfordii* and *T. hypoglaucum*. Therefore, we collected *T. wilfordii* and *T. hypoglaucum* from several producing regions to study intraspecific and interspecific variation and develop potential DNA barcodes.

### 4.1 Genomic structural characteristics and the discovery of molecular markers in *Tripterygium*

According to the structural comparison of the complete chloroplast genome, we found that the sequence length of *T. hypoglaucum* (156,936–157,061 bp) was longer than that of *T. wilfordii* (156,692–156,699 bp), and the length of *T. regelii* (159,144 bp) was longest. The primary reason for the variation in chloroplast genome size was the expansion and contraction of IR regions (Wang et al., 2008). In this study, the IR region of *T. hypoglaucum* was slightly larger than that of *T. wilfordii*. It appeared that the interspecific difference in the expansion and contraction of IR regions was not significant. However, we could still observe the presence of some specific features. For example, the length of intervals between the gene *ndhF* and the IR/SSC boundaries was different between the three species. Two commonalities of *Tripterygium* were found in the analyses of SSRs. One was that SSRs were primarily distributed in the LSC region, followed by the SSC region, and the least in the IR regions. And most SSRs were identified in the intergenic regions, followed by introns and exons (**Supplementary Table S5**). The other was that the numbers of AAT/ATT and ACCGG/CCGGT repeats were the same among *Tripterygium*. And the number of AT/AT repeats was 3 in *T. hypoglaucum*, 1 in *T. wilfordii*, and 2 in *T. regelii*. In addition, 23 palindromic repeats, 10 dispersed repeats, and 6 tandem repeats have been found in all *T. wilfordii* samples, while a 63-bp repeat existed in all *T. hypoglaucum* samples. Therefore, the significant differences in the number of repeat elements could be used to develop potential markers to species differentiation in *Tripterygium* and even to distinguish different sources of *T. hypoglaucum*.

Moreover, the alignment showed that the length of several intergenic regions varied between *T. wilfordii* and *T. hypoglaucum*. And hotspot mutation regions have been studied based on the calculated Pi values. These variable regions were valuable for species identification.

Intraspecific differences were studied as representatives of *T. hypoglaucum* collected from different provinces. Geographically adjacent samples, such as YN1 and YN2, displayed the same assembled chloroplast genomes. However, significant differences existed in sequences from Sichuan and Guangxi provinces. As **Figure 6** showed, GX1 and GX2, both collected from Guilin City, Guangxi Province, were classified into different clades and formed a well-supported. And the AAATT/AATTT repeat was only detected in sample GX2. The same features were found in the samples from Sichuan province, with the characteristic C/G and AATCCT/AGGATT repeats detected in sample SC3. Moreover, the intervals from the gene *ndhF* to IR/SSC boundary ranged from 28 to 40 bp, and the samples from neighboring Sichuan and Yunan provinces were identical (28 bp).

### 4.2 Positive selection analysis and divergence time estimation

The evolutionary rate ratio dN/dS, which represents the ratio of nonsynonymous to synonymous substitution rates and was used to identify protein sites that experience purifying selection (dN/dS < 1), evolve neutrally (dN/dS ≈ 1), or positive selection (dN/dS > 1), was the most widely used method to infer selection pressure (Nielsen and Yang, 1998; Spielman and Wilke, 2015). In our present study, the *ndhB* gene, which was closely related to the land adaptation of photosynthesis (Joeüt et al., 2001; Martín and Sabater, 2010), was identified to undergo positive selection. And the positive selected site in the *ndhB* gene may play crucial roles in the adaptive evolution of the genus *Tripterygium*.

Molecular estimates with multiple DNA regions are used for inferring the age of lineages when paleontological data are lacking (Namgung et al., 2021). A previous study (Ma et al., 2017) only used *psbA-trnH*, *rpl32-trnL*, and *trnL-trnF*. However, we estimated the divergence time of *Tripterygium* using all coding regions of chloroplast genomes, ensuring the accuracy of divergence time estimates. As the result showed, the divergence of *Tripterygium* was most likely to occur in the late Miocene (5.99 Ma, 95% HPD = 3.11–8.68 Ma), which was consistent with the previous finding (Ma et al., 2017). In addition, *T. hypoglaucum* arose at approximately 4.05 Ma (95% HPD = 1.02–6.58 Ma), much earlier than *T. wilfordii* and *T. regelii* (0.17 Ma, 95% HPD = 0.15–0.24 Ma). It is likely that the Quaternary climatic oscillation could affect the genetic structure and geographical distribution (Ma et al., 2017; Na et al., 2022).

### 4.3 Conflict in the classification of *Tripterygium*

The distribution range of *Tripterygium* was very wide, ranging from the western Hengduan Mountains in southwestern China to the east. The uplift of Qinghai-Tibet Plateau (QTP) and the establishment of Asian monsoon climatic cycle have led to the diversity of species in the Hengduan Mountains (Ding et al., 2020). According to the previous phylogenetic tree (Ma et al., 2017) reconstructed by the combination of ITS2, *psbA-trnH*, *matK*, and *rbcL*, there was only one sample of *T. hypoglaucum*, collected from Kunming, Yunnan Province, clustered with *T. wilfordii* into a clade (PP = 0.517). Meanwhile, in another NJ tree (Zhang et al., 2016) based on the ITS2 and *psbA-trnH*, only one sample of *T. hypoglaucum* collected from Wugang, Hunan Province, clustered with other samples of *T. wilfordii*. However, our ML and BI analyses based on the complete chloroplast genomes consistently supported that *T. wilfordii* could be separated from *T. hypoglaucum* (BS/PP = 100/1). In addition, as the sample size of *T. regelii* was limited, it was hard to determine the sister relationship between *T. wilfordii* and *T. regelii*. The convergent sequence evolution and incomplete lineage sorting may lead to this divergence (Rokas et al., 2003).

In addition, we also considered whether the distribution of *Tripterygium* was affected by altitude, as the samples of *T. wilfordii* we collected were all from low-altitude areas (< 500 m). Therefore, more samples and nuclear genes as well as more robust phylogenetic methods and morphological and geographic evidence should be applied to resolve the taxonomic controversy of *Tripterygium* in further research.

## 5 Conclusion

In this study, we assembled 12 chloroplast genomes of *Tripterygium* and presented the comparison to reveal the interspecific difference and intraspecific variation. The characteristics of IR boundary and repeat elements used for species differentiation were proposed. And variable regions were detected by the analyses of multiple alignment and Pi calculation. One site with positive selection in the *ndhB* gene was found by adaptive evolution analysis of *Tripterygium*. And the molecular dating analysis suggested that the split within *Tripterygium* may be traced back to the late Miocene (5.99 Ma, 95% HPD = 3.11–8.68 Ma). Phylogenetic analyses supported that *T. wilfordii* and *T. hypoglaucum* were two distinct species with a high support and resolution, laying the foundation on the further study of controversial taxonomy and evolution process.

## Data availability statement

The data presented in the study are deposited in the GenBank repository, accession number OR426549-52 and OR426554-60.

## Author contributions

K-LX: Writing – original draft. Z-MZ: Writing – review & editing. W-LF: Writing – review & editing. Y-DW: Writing – review & editing. H-YJ: Writing – review & editing. FW: Writing – review & editing. S-CM: Writing – review & editing, Supervision.

## References

[B1] Bankevich A. Nurk S. Antipov D. Gurevich A. A. Dvorkin M. Kulikov A. S.. (2012). SPAdes: a new genome assembly algorithm and its applications to single-cell sequencing. J. Comput. Biol. 19, 455–477. doi: 10.1089/cmb.2012.0021 22506599 PMC3342519

[B2] Benson G. (1999). Tandem repeats finder: a program to analyze DNA sequences. Nucleic Acids Res. 27, 573–580. doi: 10.1093/nar/27.2.573 9862982 PMC148217

[B3] Brinker A. M. Ma J. Lipsky P. E. Raskin I. (2007). Medicinal chemistry and pharmacology of genus *Tripterygium* (Celastraceae). Phytochemistry 68, 732–766. doi: 10.1016/j.phytochem.2006.11.029 17250858 PMC3867260

[B4] Capella-Gutiérrez S. Silla-Martínez J. M. Gabaldón T. (2009). trimAl: a tool for automated alignment trimming in large-scale phylogenetic analyses. Bioinformatics 25, 1972–1973. doi: 10.1093/bioinformatics/btp348 19505945 PMC2712344

[B5] Chen S. F. Zhou Y. Q. Chen Y. R. Gu J. (2018). fastp: an ultra-fast all-in-one FASTQ preprocessor. Bioinformatics 34, i884–i890. doi: 10.1093/bioinformatics/bty560 30423086 PMC6129281

[B6] Chen Y. L. Liu X. Qu X. Y. Yao Y. Y. Li N. Liang X. M.. (2017). Studies on difference of chemical compositions in plant species of *Tripterygium* genus. China J. Chin. Mater. Med. 42, 319–325. doi: 10.19540/j.cnki.cjcmm.20161222.011 28948738

[B7] Darling A. E. Mau B. Perna N. T. (2010). progressiveMauve: multiple genome alignment with gene gain, loss and rearrangement. PloS One 5, e11147. doi: 10.1371/journal.pone.0011147 20593022 PMC2892488

[B8] Ding W. N. Ree R. H. Spicer R. A. Xing Y. W. (2020). Ancient orogenic and monsoon-driven assembly of the world’s richest temperate alpine flora. Science 369, 578–581. doi: 10.1126/science.abb4484 32732426

[B9] Drummond A. J. Suchard M. A. Xie D. Rambaut A. (2012). Bayesian phylogenetics with BEAUti and the BEAST 1.7. Mol. Biol. Evol. 29, 1969–1973. doi: 10.1093/molbev/mss075 22367748 PMC3408070

[B10] Fan W. B. Wu Y. Yang J. Shahzad K. Li Z. H. (2018). Comparative chloroplast genomics of dipsacales species: insights into sequence variation, adaptive evolution, and phylogenetic relationships. Front. Plant Sci. 9. doi: 10.3389/fpls.2018.00689 PMC5974163 29875791

[B11] Frazer K. A. Pachter L. Poliakov A. Rubin E. M. Dubchak I. (2001). VISTA: computational tools for comparative genomics. Nucleic Acids Res. 32, W273–W279. doi: 10.1093/nar/gkh458 PMC441596 15215394

[B12] Ge Y. Xie H. Li S. Jin B. Hou J. Zhang H.. (2013). Treatment of diabetic nephropathy with *Tripterygium wilfordii* Hook F extract: a prospective, randomized, controlled clinical trial. J. Transl. Med. 31, 134. doi: 10.1186/1479-5876-11-134 PMC3670993 23725518

[B13] Guo L. Duan L. Liu K. Liu E. H. Li P. (2014). Chemical comparison of *Tripterygium wilfordii* and *Tripterygium hypoglaucum* based on quantitative analysis and chemometrics methods. J. Pharm. Biomed. Anal. 95, 220–228. doi: 10.1016/j.jpba.2014.03.009 24694566

[B14] Guo M. Pang X. Xu Y. Jiang W. Liao B. Yu J.. (2022). Plastid genome data provide new insights into the phylogeny and evolution of the genus Epimedium. J. Adv. Res. 36, 175–185. doi: 10.1016/j.jare.2021.06.020 35127172 PMC8799909

[B15] Joeüt T. Cournac L. Horvath E. M. Medgyesy P. Peltier G. (2001). Increased sensitivity of photosynthesis to antimycin a induced by inactivation of the chloroplast NDHB gene. Evidence for a participation of the NADH-dehydrogenase complex to cyclic electron flow around photosystem I1. Plant Physiol. 125, 1919–1929. doi: 10.1104/pp.125.4.1919 11299371 PMC88847

[B16] Kalyaanamoorthy S. Minh B. Q. Wong T. K. F. von Haeseler A. Jermiin L. S. (2017). ModelFinder: fast model selection for accurate phylogenetic estimates. Nat. Methods 14, 587–589. doi: 10.1038/nmeth.4285 28481363 PMC5453245

[B17] Katoh K. Standley D. M. (2013). MAFFT multiple sequence alignment software version 7: improvements in performance and usability. Mol. Biol. Evol. 30, 772–780. doi: 10.1093/molbev/mst010 23329690 PMC3603318

[B18] Kurtz S. Choudhuri J. V. Ohlebusch E. Schleiermacher C. Stoye J. Giegerich R. (2001). REPuter: the manifold applications of repeat analysis on a genomic scale. Nucleic Acids Res. 29, 4633–4642. doi: 10.1093/nar/29.22.4633 11713313 PMC92531

[B19] Kurtz S. Phillippy A. Delcher A. L. Smoot M. Shumway M. Antonescu C.. (2004). Versatile and open software for comparing large genomes. Genome Biol. 5, R12. doi: 10.1186/gb-2004-5-2-r12 14759262 PMC395750

[B20] Law S. K. Simmons M. P. Techen N. Khan I. A. He M. F. Shaw P. C.. (2011). Molecular analyses of the Chinese herb Leigongteng (*Tripterygium wilfordii* Hook.f.). Phytochemistry 72, 21–26. doi: 10.1016/j.phytochem.2010.10.015 21094504

[B21] Letunic I. Bork P. (2021). Interactive Tree Of Life (iTOL) v5: an online tool for phylogenetic tree display and annotation. Nucleic Acids Res. 49, W293–W296. doi: 10.1093/nar/gkab301 33885785 PMC8265157

[B22] Li H. Guo Q. Xu L. Gao H. Liu L. Zhou X. (2023). CPJSdraw: analysis and visualization of junction sites of chloroplast genomes. PeerJ 11, e15326. doi: 10.7717/peerj.15326 37193025 PMC10182761

[B23] Liu W. S. Guo B. L. Huang W. H. Si J. P. (2007). RAPD analysis for genetic relationship and diversity of three species of genus *Tripterygium* . China J. Chin. Mater. Med. 32, 1615–1621. doi: 10.3321/j.issn:1001-5302.2007.16.002 18027649

[B24] Lv H. Jiang L. Zhu M. Li Y. Luo M. Jiang P.. (2019). The genus *Tripterygium*: A phytochemistry and pharmacological review. Fitoterapia 137, 104190. doi: 10.1016/j.fitote.2019.104190 31163199

[B25] Ma B. Hu T. Li P. Yuan Q. Lin Z. Tu Y.. (2017). Phylogeographic and phylogenetic analysis for *Tripterygium* species delimitation. Ecol. Evol. 7, 8612–8623. doi: 10.1002/ece3.3344 29075476 PMC5648662

[B26] Martín M. Sabater B. (2010). Plastid ndh genes in plant evolution. Plant Physiol. Biochem. 48, 636–645. doi: 10.1016/j.plaphy.2010.04.009 20493721

[B27] Na L. Wang D. Chen Y. L. Yao Y. Y. Zhang X. M. Li S. R. (2022). Phylogeography of *Tripterygium* inferred based on ITS2 DNA sequence variation. Zhong Cao Yao 53, 5476–5483. doi: 10.7501/j.issn.0253-2670.2022.17.024

[B28] Namgung J. Do H. D. K. Kim C. Choi H. J. Kim J. H. (2021). Complete chloroplast genomes shed light on phylogenetic relationships, divergence time, and biogeography of Allioideae (Amaryllidaceae). Sci. Rep. 11, 3262. doi: 10.1038/s41598-021-82692-5 33547390 PMC7865063

[B29] Nguyen L. T. Schmidt H. A. von Haeseler A. Minh B. Q. (2015). IQ-TREE: a fast and effective stochastic algorithm for estimating maximum-likelihood phylogenies. Mol. Biol. Evol. 32, 268–274. doi: 10.1093/molbev/msu300 25371430 PMC4271533

[B30] Nielsen R. Yang Z. (1998). Likelihood models for detecting positively selected amino acid sites and applications to the HIV-1 envelope gene. Genetics 148, 929–936. doi: 10.1093/genetics/148.3.929 9539414 PMC1460041

[B31] Palmer J. D. (1985). Comparative organization of chloroplast genomes. Annu. Rev. Genet. 19, 325–354. doi: 10.1146/annurev.ge.19.120185.001545 3936406

[B32] Rambaut A. Drummond A. J. Xie D. Baele G. Suchard M. A. (2018). Posterior summarization in Bayesian phylogenetics using Tracer 1.7. Syst. Biol. 67, 901–904. doi: 10.1093/sysbio/syy032 29718447 PMC6101584

[B33] Rokas A. Williams B. L. King N. Carroll S. B. (2003). Genome-scale approaches to resolving incongruence in molecular phylogenies. Nature 425, 798–804. doi: 10.1038/nature02053 14574403

[B34] Ronquist F. Teslenko M. van der Mark P. Ayres D. L. Darling A. Höhna S.. (2012). MrBayes 3.2: efficient Bayesian phylogenetic inference and model choice across a large model space. Syst. Biol. 61, 539–542. doi: 10.1093/sysbio/sys029 22357727 PMC3329765

[B35] Rozas J. Ferrer-Mata A. Sánchez-DelBarrio J. C. Guirao-Rico S. Librado P. Ramos-Onsins S. E.. (2017). DnaSP 6: DNA sequence polymorphism analysis of large data sets. Mol. Biol. Evol. 34, 3299–3302. doi: 10.1093/molbev/msx248 29029172

[B36] Shi L. Chen H. Jiang M. Wang L. Wu X. Huang L.. (2019). CPGAVAS2, an integrated plastome sequence annotator and analyzer. Nucleic Acids Res. 47, W65–W73. doi: 10.1093/nar/gkz345 31066451 PMC6602467

[B37] Spielman S. J. Wilke C. O. (2015). The relationship between dN/dS and scaled selection coefficients. Mol. Biol. Evol. 32, 1097–1108. doi: 10.1093/molbev/msv003 25576365 PMC4379412

[B38] Tao X. Davis L. S. Lipsky P. E. (1991). Effect of an extract of the Chinese herbal remedy *Tripterygium wilfordii* Hook F on human immune responsiveness. Arthritis Rheumatol. 34, 1274–1281. doi: 10.1002/art.1780341011 1930317

[B39] Thiel T. Michalek W. Varshney R. Graner A. (2003). Exploiting EST databases for the development and characterization of gene-derived SSR-markers in barley (*Hordeum vulgare* L.). Theor. Appl. Genet. 106, 411–422. doi: 10.1007/s00122-002-1031-0 12589540

[B40] Tong L. Zhao Q. Datan E. Lin G. Q. Minn I. Pomper M. G.. (2021). Triptolide: reflections on two decades of research and prospects for the future. Nat. Prod. Rep. 38, 843–860. doi: 10.1039/d0np00054j 33146205

[B41] Wang R. J. Cheng C. L. Chang C. C. Wu C. L. Su T. M. Chaw S. M. (2008). Dynamics and evolution of the inverted repeat-large single copy junctions in the chloroplast genomes of monocots. BMC Evol. Biol. 8, 36. doi: 10.1186/1471-2148-8-36 18237435 PMC2275221

[B42] Wang Q. Meng J. Dong A. Yu J. Z. Zhang G. X. Ma C. G. (2016). The genus *Tripterygium*: A phytochemistry and pharmacological review. J. Altern. Complement Med. 22, 496–502. doi: 10.1089/acm.2016.0004 27224044

[B43] Xiong A. S. Peng R. H. Zhuang J. Gao F. Zhu B. Fu X. Y.. (2009). Gene duplication, transfer, and evolution in the chloroplast genome. Biotechnol. Adv. 27, 340–347. doi: 10.1016/j.bioteChadv.2009.01.012 19472510

[B44] Xu X. M. Wei Z. Sun J. Z. Zhao Q. F. Lu Y. Wang Z. L.. (2023). Phylogeny of Leontopodium (Asteraceae) in China—with a reference to plastid genome and nuclear ribosomal DNA. Front. Plant Sci. 14. doi: 10.3389/fpls.2023.1163065 PMC10425225 37583593

[B45] Yan L. Wang H. Huang X. Li Y. Yue Y. Wang Z.. (2022). Chloroplast genomes of genus *Tilia*: comparative genomics and molecular evolution. Front. Genet. 13. doi: 10.3389/fgene.2022.925726 PMC9305825 35873491

[B46] Yang Z. (2007). PAML 4: phylogenetic analysis by maximum likelihood. Mol. Biol. Evol. 24, 1586–1591. doi: 10.1093/molbev/msm088 17483113

[B47] Zhang X. M. Li N. Yao Y. Y. Liang X. M. Qu X. Y. Liu X.. (2016). Identification of species in *Tripterygium* (Celastraceae) based on DNA barcoding. Biol. Pharm. Bull. 39, 1760–1766. doi: 10.1248/bpb.b15-00956 27601081

[B48] Zheng S. Poczai P. Hyvönen J. Tang J. Amiryousefi A. (2020). Chloroplot: an online program for the versatile plotting of organelle genomes. Front. Genet. 11. doi: 10.3389/fgene.2020.576124 PMC7545089 33101394

