# Supplementary figures and images for: Comparative analyses of complete chloroplast genomes reveal interspecific difference and intraspecific variation of Tripterygium genus
Source: Front Plant Sci. 2024 Jan 9;14:1288943. doi: 10.3389/fpls.2023.1288943 (PMC10803662; doi:10.3389/fpls.2023.1288943)

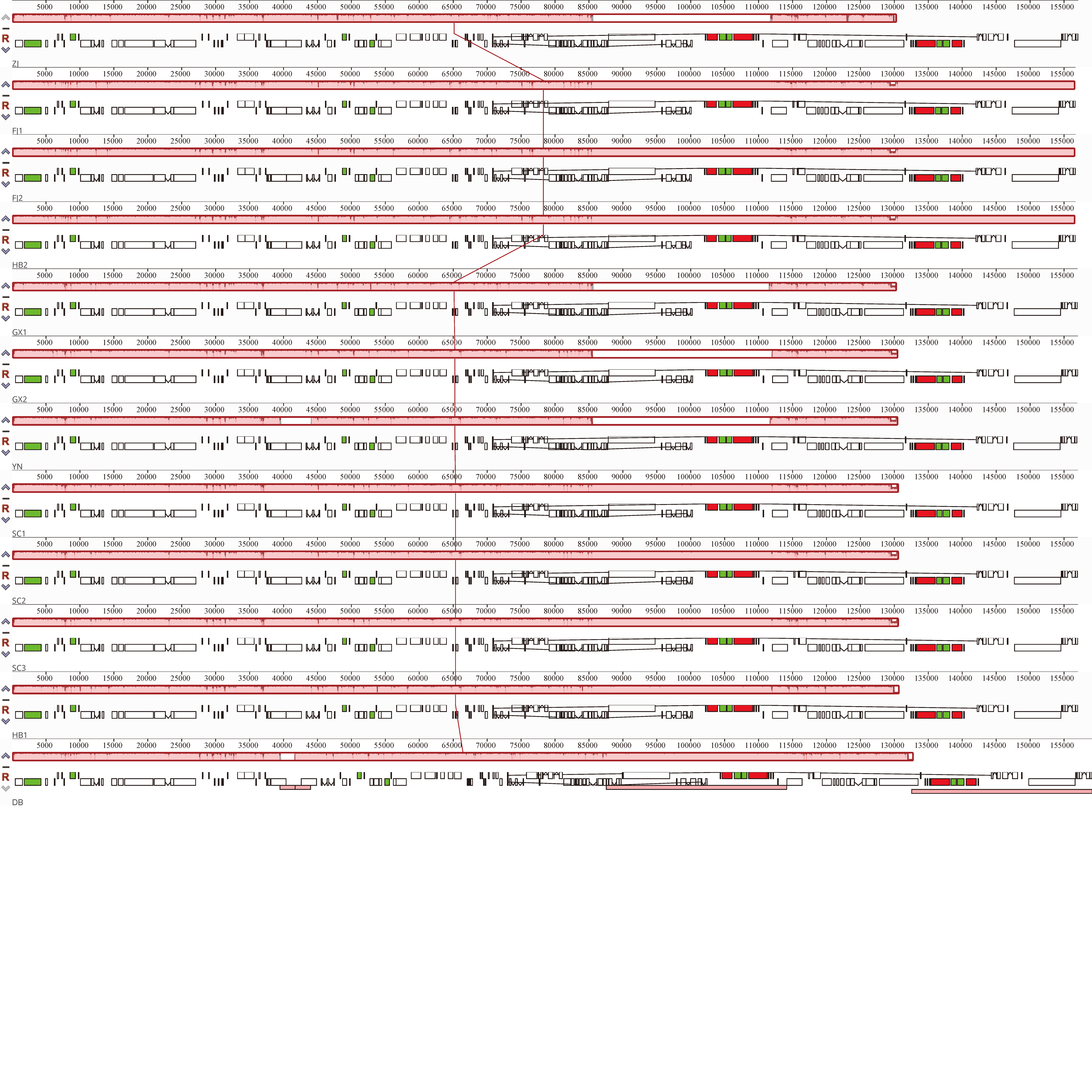

Supplement: Supplementary file 1 [file Image_1.tif]
