# Supplementary material for: Comparative analyses of complete chloroplast genomes reveal interspecific difference and intraspecific variation of Tripterygium genus
Source: Front Plant Sci. 2024 Jan 9;14:1288943. doi: 10.3389/fpls.2023.1288943 (PMC10803662; doi:10.3389/fpls.2023.1288943)
Supplement: Supplementary file 2 [file Table_1.docx]

**Supplementary Table 1 Collection information and GenBank accession number of samples.**

| Species | Sample ID | Accession numbers | Location |  | Longitude and latitude | Altitude (m) | Collection times |
| --- | --- | --- | --- | --- | --- | --- | --- |
| *T*. *wilfordii* | ZJ | OR426549 | Shaoxing, Zhejiang | cultivated | 119.7925/29.3394 | 420.1 | 2022.10 |
|  | FJ1 | OR426550 | Sanming, Fujian | wild | 117.0767/26.8453 | 344.8 | 2022.10 |
|  | FJ2 | OR426551 | Sanming, Fujian | wild | 117.1981/26.8864 | 350.2 | 2022.10 |
|  | HB2 | OR426552 | Huangshi, Hubei | cultivated | 115.06/29.92 | 103 | 2023.05 |
| *T. hypoglaucum* | GX1 | OR426554 | Guilin, Guangxi | wild | 110.1947/25.8047 | 1090.9 | 2022.10 |
|  | GX2 | OR426555 | Guilin, Guangxi | wild | 110.7394/25.2144 | 828.2 | 2022.10 |
|  | YN1 | OR426556 | Chuxiong, Yunnan | wild | 101.6058/24.9047 | 2114.0 | 2022.11 |
|  | YN2 | OR426556 | Chuxiong, Yunnan | wild | 101.4544/24.9197 | 1918.5 | 2022.11 |
|  | SC1 | OR426557 | Panzhihua, Sichuan | cultivated | 101.9567/27.0522 | 1813.1 | 2022.11 |
|  | SC2 | OR426558 | Panzhihua, Sichuan | wild | 102.0081/26.9944 | 1828.4 | 2022.11 |
|  | SC3 | OR426559 | Liangshan, Sichuan | wild | 102.1356/27.7294 | 1822.8 | 2022.11 |
|  | HB1 | OR426560 | Enshi, Hubei | wild | 111.3000/29.8833 | 1118.4 | 2023.05 |
