# Supplementary material for: Comparative analyses of complete chloroplast genomes reveal interspecific difference and intraspecific variation of Tripterygium genus
Source: Front Plant Sci. 2024 Jan 9;14:1288943. doi: 10.3389/fpls.2023.1288943 (PMC10803662; doi:10.3389/fpls.2023.1288943)
Supplement: Supplementary file 3 [file Table_2.docx]

**Supplementary Table 2 Gene annotated in the chloroplast genomes.**

| Category for genes | Group of genes | Gene names |
| --- | --- | --- |
| Self-replication | rRNA genes | *rrn16*, *rrn23*, *rrn4.5*, *rrn5* |
|  | tRNA genes | *trnI-CAU*, *trnL-CAA*, *trnV-GAC*, *trnI-GAU*, *trnA-UGC*, *trnR-ACG*, *trnN-GUU*, *trnL-UAG*, *trnP-UGG*, *trnW-CCA*, *trnM-CAU*, *trnV-UAC*, *trnF-GAA*, *trnL-UAA*, *trnT-UGU*, *trnS-GGA*, *trnfM-CAU*, *trnG-GCC*, *trnS-UGA, trnT-GGU*, *trnE-UUC*, *trnY-GUA*, *trnD-GUC*, *trnC-GCA*, *trnR-UCU*, *trnG-UCC*, *trnS-GCU*, *trnQ-UUG*, *trnK-UUU*, *trnH-GUG* |
|  | Small subunit of ribosome | *rps19*, *rps7*, *rps15*, *rps3*, *rps8*, *rps11*, *rps12*, *rps18*, *rps4*, *rps14*, *rps2*, *rps16* |
|  | Large subunit of ribosome | *rpl2*, *rpl23*, *rpl32*, *rpl22*, *rpl16*, *rpl14*, *rpl36*, *rpl20*, *rpl33* |
|  | DNA dependent RNA polymerase | *rpoA*, *rpoB*, *rpoC1*, *rpoC2* |
| Genes for photosynthesis | Subunits of NADH-dehydrogenase | *ndhA*, *ndhB*, *ndhC*, *ndhD*, *ndhE*, *ndhF*, *ndhG*, *ndhH*, *ndhI*, *ndhJ*, *ndhK* |
|  | Subunits of photosystem I | *psaA*, *psaB*, *psaC*, *psaI*, *psaJ* |
|  | Subunits of photosystem II | *psbA*, *psbB*, *psbC*, *psbD*, *psbE*, *psbF*, *psbH*, *psbI*, *psbJ*, *psbK*, *psbM*, *psbN*, *psbT*, *psbZ* |
|  | Subunits of cytochrome b/f complex | *petA*, *petB*, *petD*, *petG*, *petL*, *petN* |
|  | Subunits of ATP synthase | *atpA*, *atpB*, *atpE*, *atpF*, *atpH*, *atpI* |
|  | Large subunits of rubisco | *rbcL* |
| Other genes | Maturase | *matK* |
|  | Protease | *clpP* |
|  | Envelope membrane protein | *cemA* |
|  | Subunit of acetyl-CoA-carboxylase | *accD* |
|  | C-type cytochrome synthesis gene | *ccsA* |
|  | Translational initiation factor 1 | *infA* |
| Genes of unknown function | Open Reading Frames | *ycf1*, *ycf2*, *ycf3*, *ycf4* |
