# Supplementary material for: Comparative analyses of complete chloroplast genomes reveal interspecific difference and intraspecific variation of Tripterygium genus
Source: Front Plant Sci. 2024 Jan 9;14:1288943. doi: 10.3389/fpls.2023.1288943 (PMC10803662; doi:10.3389/fpls.2023.1288943)
Supplement: Supplementary file 5 [file Table_4.docx]

**Supplementary Table 4 Likelihood ratio test (LRT) of the variable ω ratio under different models.**

| gene | comparisons | 2Δl | df | p |
| --- | --- | --- | --- | --- |
| *atpA* | M1 vs M2 | 1.269156 | 2 | 5.302e-1 |
|  | M7 vs M8 | 1.281346 | 2 | 5.269e-1 |
| *ccsA* | M1 vs M2 | 3.744892 | 2 | 1.537e-1 |
|  | M7 vs M8 | 3.744896 | 2 | 1.537e-1 |
| *matK* | M1 vs M2 | 1.23201 | 2 | 5.401e-1 |
|  | M7 vs M8 | 1.23201 | 2 | 5.401e-1 |
| *ndhB* | M1 vs M2 | 11.460462 | 2 | 3.246e-3 |
|  | M7 vs M8 | 11.274086 | 2 | 3.563e-3 |
| *rpl20* | M1 vs M2 | 0.724162 | 2 | 6.962e-1 |
|  | M7 vs M8 | 0.724162 | 2 | 6.962e-1 |
| *rpoA* | M1 vs M2 | 0.875414 | 2 | 6.455e-1 |
|  | M7 vs M8 | 0.882594 | 2 | 6.432e-1 |
