# Supplementary material for: Comparative analyses of complete chloroplast genomes reveal interspecific difference and intraspecific variation of Tripterygium genus
Source: Front Plant Sci. 2024 Jan 9;14:1288943. doi: 10.3389/fpls.2023.1288943 (PMC10803662; doi:10.3389/fpls.2023.1288943)
Supplement: Supplementary file 6 [file Table_5.docx]

**Supplementary Table 5 Distributions of SSRs in the *Tripterygium* chloroplast genomes.**

| Species | Sample ID | Total number | in LSC | in SSC | in IRs | In IGS | In exon | In intron |
| --- | --- | --- | --- | --- | --- | --- | --- | --- |
| *T*. *wilfordii* | ZJ | 59 | 42 | 12* | 6* | 31 | 15* | 13 |
|  | FJ1 | 59 | 42 | 12* | 6* | 31 | 15* | 13 |
|  | FJ2 | 59 | 42 | 12* | 6* | 31 | 15* | 13 |
|  | HB2 | 59 | 42 | 12* | 6* | 31 | 15* | 13 |
| *T. hypoglaucum* | GX1 | 59 | 42 | 13 | 4 | 34 | 14 | 11 |
|  | GX2 | 57 | 43 | 12 | 2 | 32 | 14 | 11 |
|  | YN1 | 58 | 44 | 10 | 4 | 33 | 15* | 10 |
|  | YN2 | 58 | 44 | 10 | 4 | 33 | 15* | 10 |
|  | SC1 | 58 | 45 | 11 | 2 | 34 | 14 | 10 |
|  | SC2 | 58 | 45 | 11 | 2 | 34 | 14 | 10 |
|  | SC3 | 57 | 43 | 12 | 2 | 32 | 15 | 10 |
|  | HB1 | 59 | 45 | 12 | 2 | 34 | 14 | 11 |
| *T. regelii* | DB | 57 | 45 | 10 | 2 | 31 | 14 | 12 |
| * A SSR partially in this region | | | | | | | | |
